# A method for isolation of cone photoreceptors from adult zebrafish retinae

**DOI:** 10.1186/s12868-016-0307-2

**Published:** 2016-11-07

**Authors:** Antonino Glaviano, Andrew J. Smith, Alfonso Blanco, Sarah McLoughlin, Maria L. Cederlund, Theresa Heffernan, Beata Sapetto-Rebow, Yolanda Alvarez, Jun Yin, Breandán N. Kennedy

**Affiliations:** UCD School of Biomolecular & Biomedical Science, UCD Conway Institute, University College Dublin, Dublin, D04 V1W8 Ireland

**Keywords:** Cone photoreceptors, Flow cytometry, Cell sorting, RNA, Zebrafish

## Abstract

**Background:**

Cone photoreceptors are specialised sensory retinal neurons responsible for photopic vision, colour perception and visual acuity. Retinal degenerative diseases are a heterogeneous group of eye diseases in which the most severe vision loss typically arises from cone photoreceptor dysfunction or degeneration. Establishing a method to purify cone photoreceptors from retinal tissue can accelerate the identification of key molecular determinants that underlie cone photoreceptor development, survival and function. The work herein describes a new method to purify enhanced green fluorescent protein (EGFP)-labelled cone photoreceptors from adult retina of Tg(3.2*gnat2:EGFP*) zebrafish.

**Results:**

Methods for dissecting adult zebrafish retinae, cell dissociation, cell sorting, RNA isolation and RNA quality control were optimised. The dissociation protocol, carried out with ~30 retinae from adult zebrafish, yielded approximately 6 × 10^6^ cells. Flow cytometry cell sorting subsequently distinguished 1 × 10^6^ EGFP^+^ cells and 4 × 10^6^ EGFP^−^ cells. Electropherograms confirmed downstream isolation of high-quality RNA with RNA integrity number (RIN) >7.6 and RNA concentration >5.7 ng/µl obtained from both populations. Reverse Transcriptase-PCR confirmed that the EGFP-positive cell populations express known genetic markers of cone photoreceptors that were not expressed in the EGFP-negative cell population whereas a rod opsin amplicon was only detected in the EGFP-negative retinal cell population.

**Conclusions:**

This work describes a valuable adult zebrafish cone photoreceptor isolation methodology enabling future identification of cone photoreceptor-enriched genes, proteins and signalling networks responsible for their development, survival and function. In addition, this advancement facilitates the identification of novel candidate genes for inherited human blindness.

## Background

The vertebrate retina is a light-sensitive tissue lining the inner-surface of the eye, which is organised into cell layers with microcircuits working in parallel and together to encode visual information. Light is captured by photoreceptors in the retina whose circuit of afferent outputs constitutes the major sensory inputs to the brain [1]. Photoreceptors are specialised sensory retinal neurons that enable images of the external environment to be captured. Cone and rod photoreceptors are structurally similar, however, cones function in relatively bright light, or photopic vision, whereas rods are specialised to function in dim light, or scotopic vision [2].

Retinal degenerative diseases are a heterogeneous group of eye diseases in which there is a slow but progressive loss of photoreceptors, resulting in blindness [3]. One in 2000 people present with an inherited retinal degeneration caused by mutation in one of more than 200 causative genes, culminating in more than 3.2 million people blind due to retinal degenerations [4]. Loss or dysfunction of cone photoreceptors during retinal degeneration is a principal reason for severe human blindness [5]. Even when rod photoreceptors die first in inherited retinitis pigmentosa, the subsequent death of cones has the most significant impact on vision [6–8].

The majority of advances in our understanding of photoreceptor biology has arisen from characterisation of rod photoreceptors as a result of the extensive use of nocturnal rodent models, which contain >97% rod and <3% cone photoreceptors [9, 10]. For rods, an extensive array of genetic regulators of photoreceptor morphogenesis, phototransduction, visual cycle, lipid and intermediary metabolism, ciliary trafficking, cell signalling, pre-mRNA splicing, intracellular transport, innate immunity and phagocytosis are known and many have been identified as causative genes in inherited forms of blindness [8]. In contrast, the study of cone photoreceptors has lagged behind. The human retina contains ~6 million cones and ~120 million rods [11, 12]. Notably, however, cone photoreceptors in primates are highly concentrated into a specialised central area of the retina known as the macula [13] to which light is focused for most daylight and fine-detail tasks e.g. colour vision, reading and driving [14].

One impediment to cone research is the lack of cone-enriched vertebrate models amenable to laboratory investigation [15]. Alternative diurnal models that possess cone-rich retinas include: pigs (~15–20% cones), ground squirrels (~90% cones) and chickens (~65% cones); but these present individual difficulties for experimentation or maintenance [15–18]. Cone-rich transgenic models such as the *Nrl* ^−/−^ mouse are available, and provide a model for the investigation of cone function and cone-specific genetic disease. However, questions remain as to the pedigree of the cones in such strains, as the S-cones degenerate resulting in enhanced S-cone syndrome [19, 20]. Similarly, there is no human cell line that recapitulates the specialised architecture of cone photoreceptor morphology. For example, the immortalised mouse retinal cell line 661 W has been shown to express SV40 T antigen, blue and green cone pigments, transducin, and cone arrestin, however, these cells do not express rod-specific antigens, such as opsin and arrestin or rod- and cone-specific proteins such as phosducin, peripherin/rds, and ROM1 [21].

Purification of rod and cone retinal photoreceptors is a priority of many research groups. High-quality RNA for microarray analysis has been obtained from flow cytometry sorted mouse rod photoreceptors [22–24] as well as from magnetic associated cell-sorted (MACS) rod photoreceptors [25, 26]. A flow cytometry cell sorting methodology based on the strong light scattering properties of compacted heterochromatin allowed sorting of adult mice rod photoreceptors [27, 28].

Furthermore, flow cytometry sorted mouse photoreceptors [29, 30], embryonic mouse photoreceptor precursors [31], and retinal progenitor cells derived from human induced-pluripotent stem cells [32] have been isolated. Kaewkhaw et al. [33] recently purified developing human photoreceptors from organoid 3D cultures systems producing global gene expression profiles delineating gene regulatory networks underlying photoreceptor differentiation.

In contrast, purification of cone photoreceptors has only been reported in a few studies. Cone photoreceptors were isolated from carp with a stepwise Percoll gradient [34, 35], from which suppression subtractive hybridization [36] identified genes expressed preferentially in cones or rods [37]. High-quality RNA was recently extracted from flow cytometry sorted GFP-positive cone photoreceptors of transgenic Tg(3.2*gnat2*:EGFP) and *pde6c* mutant larval zebrafish for gene expression analysis [38, 39].

Zebrafish offer an excellent opportunity to advance our understanding of photoreceptor biology [40]. Similar to the mammalian eye, the cone-rich retina of the diurnal zebrafish consists of seven major cell classes, and its photoreceptor cells display the same gross morphological characteristics as mouse or human cells [41]. Moreover, mutations in zebrafish genes have provided models for several human genetic disorders [38, 42–45].

Herein we describe a reproducible method to purify adult cone photoreceptors from adult Tg(*3.2gnat2:EGFP*) [38]. Methods for retinal dissection, cell dissociation, cell sorting and isolation of high quality/quantity RNA were optimised.

## Methods

**Fig. 1:**
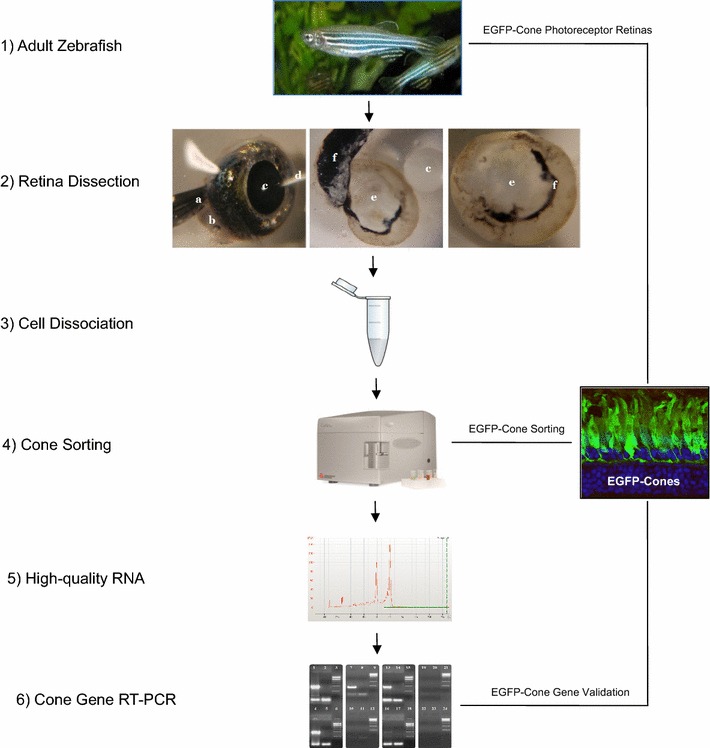
Schematic overview of the main steps to purify adult zebrafish cone photoreceptors. **1** Adult Tg(3.2TaCP:EGFP) zebrafish were enucleated using sterilised forceps. **2** Adult zebrafish were enucleated using sterilised forceps stored in DEPC-PBS (*a*) from the optic nerve (*b*) and then kept in cold DEPC-PBS. Cornea was pierced (*c*) and lens removed with a needle (*d*) to facilitate the onset of the dissection (*left picture*). EGF-cone photoreceptor retinas (*e*) were dissected from the eyes (*c*) and the retinal pigment epithelium (RPE) (*f*) with sterilised forceps (*middle picture*). Most of the RPE (*f*) was removed from the dissected retinas (*e*) (*right picture*). **3** Retinal cells were dissociated with trypsin. **4** EGFP-positive cone photoreceptors were purified by flow-cytometry. **5** High-quality RNA was obtained from sorted-GFP-positive cone photoreceptors. **6** Reverse transcriptase-PCR (RT-PCR) validated the purification of cone photoreceptors

The methodology designed to purify adult cone photoreceptors from zebrafish can be summarised with the following main steps: *Retina Dissection*, *Cell Dissociation*, *Flow Cytometry*-*Cell Sorting*, *RNA Isolation* and *RNA Quality/Quantity Control*. A schematic overview of these main steps is shown in Fig. 1.

### Retina dissection

- Adult zebrafish were euthanised in 2 mg/ml of Benzocaine (Sigma) for approximately 10 s.
- Eyes were enucleated with sterilised forceps and kept in ice-cold DEPC-PBS.
- The optic nerve was used to hold down the eye while the cornea was perforated using a fine Tungsten needle. Diamond jeweller’s needles were used to peel off corneal and scleral tissue (Fig. 1).
- The neural retina was dissected free of retinal pigment epithelium (RPE) and the lens was removed and kept in cold DEPC-PBS (Fig. 1).

### Cell dissociation

- 30 retinae were collected into 2 sterile Eppendorf tubes.
- Chemical retinal dissociation was initiated by the addition of 900 μl of 0.05% Trypsin (Gibco) diluted in DEPC-PBS, and were incubated at 37 °C for 10 min.
- Suspensions were then mechanically triturated using a P-200 followed by a 0.8 × 40 mm surgical needle and a 0.5 × 16 mm needle.
- 100 μl Trypsin inhibitor (10 mg/ml, final concentration 1 mg/ml) was then added and suspensions were centrifuged at 1200 rpm for 5 min. Supernatant was removed and the cells were resuspended in 1 ml of DEPC-PBS.
- Dissociated retinae were filtered using a 50 μm CellTrics^®^Sterile Filters (Partec) to remove aggregates and non-dissociated tissue. DEPC-PBS was initially added to the sterile filter to facilitate the filtration.
- Dissociated retinae were incubated at 4 °C for 5 min before cells were centrifuged at 1400 rpm for 6 min. Cells were resuspended in 1 ml DEPC-PBS.
- Cell number was counted using the Trypan Blue exclusion assay (Sigma) and haemocytometer (Hausser Scientific). Briefly, 5 μl of Trypan Blue was added to 5 μl of cell suspension. Following application to a haemocytometer, unstained cells (live cells) were counted in 4 sets of 16 squares, provided they were wholly in the square. The average number of cells from 4 squares was multiplied by 10^4^, then multiplied by two to correct for dilution in Trypan Blue.

### Flow cytometry analysis

**Fig. 2:**
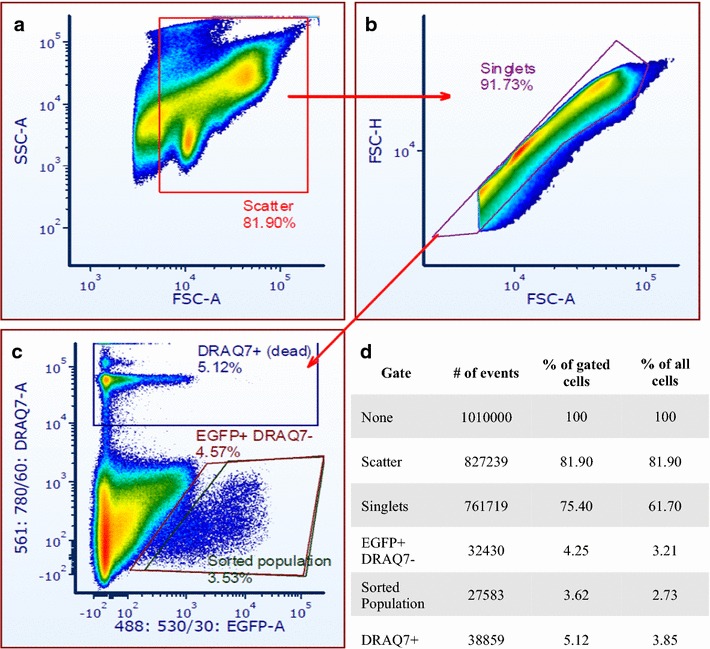
Cone photoreceptors can be easily localised based on their EGFP expression and their scatter characteristics. The figure shows the gating strategy used for the analysis and sorting of the EGFP positive cells. The first selection was only based on the scatter characteristics of cone photoreceptors (forward versus side scatter **a**, **b**) Flow cytometry cell sorting was performed using an endogenous EGFP transgene expression to enrich for cone photoreceptors with a threshold filter that removes autofluorescent cells. A drop of DRAQ7 DROP AND GO™ rapid dsDNA-dye was added to identify dead cells (excited with 633 nm laser, emission collected with the 675/25 nm Accuri C6, 665/25 nm in the Cyan ADP and the 660/20 nm in the FACSAria IIIu) which were discarded. Dead EGFP^+^ cells were discarded as they are positively labeled for DRAQ7. The gate EGFP^+^ DRAQ7^−^ (**c**, **d**) corresponds to all the EGFP positive, viable cells. The gate sorted populations is used to minimise any possible EGFP-contaminations due to autofluorescence. Table (**d**) represents one representative sorting process, and lists cell number and percentage of gated cells which were identified in the EGFP^+^ DRAQ7^−^ and the final sorted population

- Optimization of the flow cytometry cell sorting sample preparation was performed with a Beckman Coulter Cyan ADP (Summit Software) and a BD Accuri C6 (CFlow Plus Software).
- Cell sorting was performed using a BD FACSAria (BDFACSDiva Software) at 70 psi with a 70 µm nozzle.
- Quality Control (QC) of flow cytometry was carried out under standard manufacturer’s specifications.
- In order to obtain an optimised cell sorter performance the BDAccudrop (a set of QC beads) was used prior to sample sorting to ensure the maximum recovery of target cells obtained with the Fine Tune mode values higher than 95%.
- This set of QC beads is used to analyse drop formation and ensure that cells being sorted are actually included in the drops. The BDAccudrop therefore performs the important function to check that no other cells contaminates the target drop.
- EGFP excitation used 488 nm lasers and emission was collected with a 530/30 (BD Accuri C6 and BD FACSAria cell sorter) or 530/40 nm (BC Cyan ADP) band pass filters. A drop of DRAQ7 DROP AND GO™ (BioStatus), a rapid dsDNA dye with a wide emission in the infrared, was added to identify and discard dead cells.
- Cell sorting and analysis was performed on samples in freshly made DEPC-PBS. 250 µl of cold DEPC-PBS was placed in collection Eppendorf tubes to minimise mechanical cell damage during sorting.
- For better identification of target cells from tissue, logarithmic scales were used for FSC and SSC (Fig. 2). To remove non-cellular events (e.g. debris, air bubbles or electrical noise) which could interfere with analytical processes, an electronic threshold was applied on the FSC detector, to limit the acquisition of events by the flow cytometer so that only signals with an intensity greater than or equal to the threshold channel value were appropriately processed.
- The electrical pulse of aggregated events have a scatter signal with bigger area compared to single events (singlets), as well as a different area/width ratio, which allowed the removal of these aggregated events by generating regions. As shown in Fig. 2, a region named “Singlet” was drawn around the single events permitting the exclusion of aggregated events from the analysis and further sorting.
- The low-frequency EGFP cells were identified using a bivariate histogram of EGFP fluorescence versus DRAQ7. Non-transgenic wildtype and unlabeled DRAQ7 samples helped identify background autofluorescence levels.
- The gating strategy was based on EGFP fluorescence intensity. Scatter values allowed the identification and sorting of single EGFP events by flow cytometry, which corresponds to cone photoreceptors (Fig. 2). Dead EGFP cells were discarded as they are positively labeled for DRAQ7.
- Normally, 30,000–50,000 cells were sorted for RNA Analysis.

### RNA isolation and analysis

- RNA was isolated with the RNeasy Mini Kit (Qiagen) protocol [46] in order to increase the final RNA concentration; its main steps are described below.
- Sorted-cells were pelleted for 2 min at 14,000 rpm, supernatant discarded, then homogenized with 350 μl Buffer RLT + 3.5 μl β-Mercaptoethanol using a 25-gauge needle.
- Lysate transferred to a spin column was centrifuged for 2 min at 14,000 rpm. Wash steps were carried out according to manufacturer’s instruction. RNA was eluted in 30 μl RNAse free H_2_O, and stored at −80 °C.
- Eluent from 3 replicate experiments was pooled to increase RNA yield. 350 μl of 70% ethanol was added. Samples were transferred to RNeasy MinElute spin columns, centrifuged for 15 s at 10,000 rpm and the flow-through discarded.
- Wash and elution steps were performed according to the manufacturer’s instructions.
- In the last step of the protocol, RNeasy MinElute spin column was placed in a new 1.5-ml collection tube. 14 µl RNase-free water was added directly to the centre of the spin column membrane according to the manufacturer’s instructions.
- The spin column was centrifuged for 1 min at 14,000 rpm (full speed) to elute the RNA.
- RNA was quantified with an ND-1000 Spectrophotometer (Nano-Drop Technologies). RNA quality was evaluated with the Agilent 2100 Bioanalyzer (Pico-Assay).
- Approximately 79.8 ng RNA (5.7 ng/μl × 14 µl) as a total yield was obtained after each RNA extraction.

### Reverse transcriptase-PCR

- A two-step reverse transcription was performed on 1 μg total RNA with a Reverse Transcriptase cDNA synthesis system, SuperScript III^®^ VILO™ cDNA Synthesis Kit (Invitrogen) at 50 °C, after priming with random hexamers. A total volume of 20 μl cDNA was obtained after the first strand synthesis.
- cDNA was used in standard PCR reactions with 1 μl cDNA per 25 μl PCR reaction, in standard PCR conditions. OneTaq DNA Polymerase with standard buffer was used according to manufacturers guidelines. PCR was carried out for 32 cycles with extension times adjusted to 1 min per kilobase of target amplicon.
- Primers for *actb* (β-actin), *egfp* (enhanced green fluorescent protein), *rho* (rhodopsin), *gnat2* (guanine nucleotide-binding protein G protein), *pde6h* (phosphodiesterase 6H), *opn1lw2* (opsin 1 cone pigments long-wave-sensitive) and *pde6c* (phosphodiesterase 6C) were designed complementary to Expressed Sequence Tags (ESTs) identified after BLAST analysis of EST databases.
- Primer sequences with their respective melting temperatures and annealing temperatures (50–63 °C) are shown in Table 1.

**Table 1:** Primers for *actb* (β-actin), *egfp* (enhanced green fluorescent protein), *rho* (rhodopsin), *gnat2* (guanine nucleotide-binding protein G protein), *pde6h* (phosphodiesterase 6H), *opn1lw2* (opsin 1 cone pigments long-wave-sensitive) and *pde6c* (phosphodiesterase 6C) were designed complementary to Expressed Sequence Tags (ESTs) identified after BLAST analysis of EST databases

Gene	F/R	Primer sequence	Product size (bp)	Melting temperature (°C)	Annealing temperature (°C)
*actb*	F	CGAGCAGGAGATGGGAACC	100	61.0	53
	R	CAACGGAAACGCTCATTGC		58.0	
*egfp*	F	ATGGTGAGCAAGGGCGAGGAGCTGT	713	68.0	63
	R	TACAGCTCGTCCATGCCGAGAGTGATCC		70.0	
*rho*	F	AGAACCATGCCATCATGGGG	138	60.1	55
	R	GAGTGCGGGTGTAGTAGTCG		59.9	
*gnat2*	F	ACGGTCAAACTTCTGCTGCT	394	60.0	50
	R	TGCAGATTCTGTCCATTTCG		55.0	
*pde6h*	F	GACCACTCGCACCTTCAAGA	99	59.9	55
	R	ACAGTGATGTCTGTGCCGAG		60.0	
*opn1lw2*	F	TGATGGCTCTGAGGTGTCCA	105	60.5	53
	R	TCCAGTTCTTCCCTCTTGTTCA		58.9	
*pde6c*	F	CACAGTTCCTGGGATGGTCC	116	60.0	55
	R	CGGAGTGGCTTTGGTCTGAT		60.0	

It shows forward (F) and reverse (R) primer sequences with their product size (bp), melting temperatures and annealing temperatures

## Results

### Retinal dissection, cell dissociation and flow cytometry analysis

A yield of ~6 × 10^6^ cells from ~30 adult zebrafish retinae was achieved (Fig. 1). Since each tissue sample contained different cell types, as well as debris produced during the disaggregation of the tissue, the first step was identifying the population of events that encompassed the cone photoreceptors. Indeed, cones were localised based on their EGFP expression and their scatter characteristics. Once the main population was identified, a gating strategy was created in order to ensure that only single EGFP-positive cone photoreceptors were sorted. Figure 2 shows the gating strategy used for the analysis and sorting of these events. The first selection was based on their scatter characteristics (forward versus side scatter) (Fig. 2a). Aggregated events were removed by a selection of area versus height of forward scatter signals (Fig. 2b). Finally, the selection of the viable EGFP-positive cells was performed (Fig. 2c). Regions were defined by using unlabelled wildtype and single labelled DRAQ7 control sample. A backgate was applied to ensure that the selection process was correct. DRAQ7 was used as a method to identify both viable and dead EGFP^+^ cells. DRAQ7 positive cells were discarded as they correspond to non viable cells (Fig. 2c, d). Cell sorting lasted 1.5 h for 30 retinas. Among ~6 × 10^6^ dissociated cells detected from 30 retinas, ~1 × 10^6^ were EGFP^+^ and ~4 × 10^6^ EGFP^−^. Thus, ~1 × 10^6^ cells were lost during sorting. The EGFP^+^ cells sorted represented ~16% of the original population.

### RNA quantity and quality analysis

**Fig. 3:**
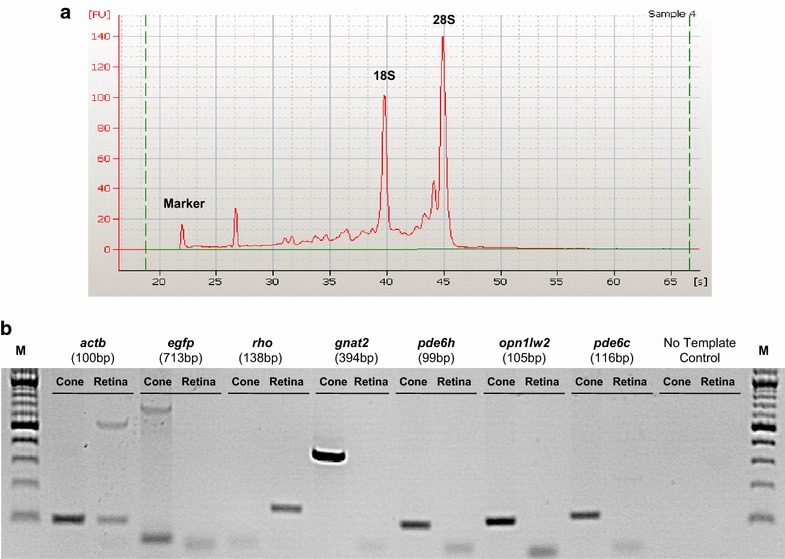
RNA Quality and Reverse Transcriptase-PCR (RT-PCR). **a** RNA quality. Electropherogram of the RNA sample (3000–4000 pg/µl) from GFP^+^ sorted cone photoreceptors using the Bioanalyzer Pico-Assay. In the final experiment, after the optimization of the techniques, high-quality RNA was obtained with the typical shape of the two ribosomal peaks (18S and 28S), and with RNA Integrity Number of 7.6, which was slightly higher than the minimum-required (7.0) for microarray analysis. **b** Reverse Transcriptase-PCR (RT-PCR) carried out for *actb*, *egfp, rho*, *gnat2, pde6h, opn1lw2* and *pde6c* in GFP-positive cone photoreceptors and GFP-negative retinas. M is NEB 2 Log DNA ladder (0.1–10.0 kb)

High-quality RNA was obtained from the sorted cone photoreceptors as demonstrated by the electropherograms with two prominent 28S and 18S ribosomal peaks (Fig. 3a). The 7.6 RNA Integrity Number (RIN) is above the 7.0 threshold recommended for dowstream transcriptomic analysis [50]. RNA concentration of 5.7 ng/µl was obtained from 1 × 10^6^ EGFP^+^ cells.

### Reverse transcriptase-PCR

RT-PCR confirmed that cone photoreceptors were highly enriched by cell sorting. Expression of *actb* (β-*actin,* a gene encoding ubiquitously expressed proteins involved in cell motility), *egfp*, *rho* (rhodopsin, a gene encoding the rod photoreceptor-specific protein rhodopsin, a G-protein coupled receptor necessary for vision in low-light conditions), *gnat2* (a gene encoding a guanine nucleotide-binding G protein, for the alpha subunit of cone transducin expressed only in cones and which couple opsin and cGMP-phosphodiesterase during phototransduction), *pde6h* (phosphodiesterase 6H, a gene encoding the inhibitory or gamma subunit of the cone-specific cGMP phosphodiesterase), *opn1lw2* (opsin 1 cone pigments long-wave-sensitive, a gene encoding for a light-absorbing visual pigment, the red cone photopigment or long-wavelength sensitive opsin protein, of the opsin gene family) and *pde6c* (phosphodiesterase 6C, a gene encoding the alpha-prime subunit of cone phosphodiesterase) are shown and described in Fig. 3b. As expected, *egfp*, *gnat2, pde6h*, *opn1lw2* and *pde6c* were expressed in EGFP^+^ cone photoreceptors, whereas *actb,* in both EGFP^+^ and EGFP^−^ neurons, and *rho* only in EGFP^−^ cells.

## Discussion

Identifying genes enriched in cone photoreceptors is an important research objective. Here, we optimised a multi-step-technique to obtain high-quality RNA from sorted-adult cone photoreceptors from a transgenic adult zebrafish line expressing EGFP specifically in cones. Optimising the flow cytometry-cell sorting technique contributed significantly to reducing RNA degradation. The simple inclusion of DEPC-PBS into collection tubes before sorting reduced cell damage and use of freshly made DEPC-PBS during sorting minimised RNA degradation.

In order to analyse and sort samples by flow cytometry, values for FSC and SSC were displayed in a logarithmic scale, as this is normally the default starting display. This allowed for the identification of different sub-populations of cells present in the retina, which were mixed with unwanted cell debris and cell fragments. Since there were multiple cell populations, different levels of auto-fluorescence were thus successfully detected. It was therefore important to change the strategy and display side scatter and fluorescence characteristics of control and EGFP samples, which ultimately allowed the identification of the extremely well-defined population of EGFP-cone photoreceptors. This improved the sorting process, and ultimately minimised RNA degradation.

Our protocol allows high-quality RNA to be obtained from sorted-adult cone photoreceptors. RNA integrity is assessed via 28S and 18S rRNA [47], and our electropherogram results demonstrate production of high-quality RNA with two clearly visible ribosomal peaks (28S and 18S) from EGFP-sorted cones. In addition, the RNA Integrity Number (RIN), an algorithm for assigning integrity values to RNA based on 28S to 18S rRNA ratios [47–49], had a value of 7.6, higher than the minimum-required 7.0 [50]. RNA yields of 5.7 ng/µl were relatively high and sufficient for downstream profiling.

RT-PCR confirmed expression of the cone specific genes *gnat2, pde6h*, *opn1lw2*, *pde6c* and *egfp,* specifically expressed in the cone photoreceptors under the 3.2 kb *gnat2* promoter fragment, but not the rod-specific gene *rho* in flow cytometry-sorted EGFP-positive photoreceptors (EGFP^+^ cells).

## Conclusions

We describe a robust methodology for the isolation of adult cone photoreceptors. This method will help advance the characterization of molecular regulators enriched in cone photoreceptors. These are a source of candidate genes for inherited blindness and therapeutic targets to overcome cone photoreceptor dysfunction. Future studies can utilise this methodology to identify cone-enriched factors by transcriptomic, proteomic, metabolomics and lipidomic analyses.

## Abbreviations

cDNA: complementary DNA
DEPC-PBS: diethylpyrocarbonate-phosphate-buffered saline
EGFP: enhanced green fluorescent protein
ESTs: expressed sequence tags
FACSAria: fluorescence-activated cell sorting aria
*FSC*: *forward*-s*cattered*
MACS: magnetic associated cell-sorting
*Nrl*: neural retina leucine zipper gene
RIN: RNA integrity number
RT-PCR: reverse transcriptase-polymerase chain reaction
QC: quality control
SSC: *side*-s*cattered*
